# Nanoplastic Particle Exposure Induces Toxicity and Amplifies Cellular Stress in THP‐1 Macrophages: Insights on Molecular Pathways and Circulating Proteome Interaction

**DOI:** 10.1002/jbt.71103

**Published:** 2026-08-31

**Authors:** Balamurali Mahalakshmi, Gobichettipalayam Balasubramaniam Maadurshni, Thittumpuram Manikandan Anjana, Jeganathan Manivannan

**Affiliations:** ^1^ Environmental Health and Toxicology Laboratory, Department of Environmental Sciences School of Life Sciences Bharathiar University Coimbatore Tamil Nadu India

**Keywords:** immune system, macrophages, nanoplastics, oxidative stress, THP‐1 cells

## Abstract

The widespread accumulation of polystyrene nanoplastic particles (NPPs) in the environment has raised significant human health concerns, specifically on immune function. However, the underlying molecular mechanisms behind NPP induced immune cell (monocyte/macrophage) toxicity and their relationship with existing pathological conditions remain largely unexplored. The current study explored the interaction of NPPs with serum proteins and composition of protein corona through proteomic analysis (LC‐HRMS/MS). Further, we investigated the immunotoxic effects on THP‐1 macrophage cells via various cellular and molecular assays, including cytotoxicity, oxidative stress, mitochondrial function, intracellular calcium homeostasis, apoptosis and stress signaling pathway assessment. This study further assessed the amplification potential of NPPs on inflammatory (TNF α and lipopolysaccharide), atherogenic (ox‐LDL) and environmental (polycyclic aromatic hydrocarbons (PAH)) stress conditions. The findings demonstrated that NPPs triggered cytotoxicity in a dose dependent manner along with excessive ROS generation, mitochondrial impairment, elevated intracellular calcium levels and apoptosis by altering BAX and BCL2 expression. We also found that NPPs activated stress signaling pathways through modulating HSP27, SAPK/JNK, p38 MAPK and c‐Jun phosphorylation. Interestingly, pathway inhibition studies confirmed the involvement of p38 MAPK, ERK and mitochondrial ROS signaling in NPP‐associated toxicity. In addition, NPPs exposure augmented inflammatory response, promoted atherogenesis (foam cell formation), amplified PAH induced toxicity. Together, these observations revealed precise adverse outcome pathways (AoP) and stress amplification effects of NPPs on macrophages and consequent toxic effects on the immune system.

## Graphical abstract

Polystyrene nanoplastic particles (NPPs) interact with serum proteins, establishing nano‐bio interactions and triggering ROS‐mediated macrophage toxicity in THP‐1 cells, characterized by oxidative stress, mitochondrial dysfunction, apoptosis and stress‐signaling. NPPs further augment inflammatory responses, promote foam cell formation and amplify PAH‐induced toxicity, collectively elucidate the mode of action underlying NPP‐induced immunotoxicity.

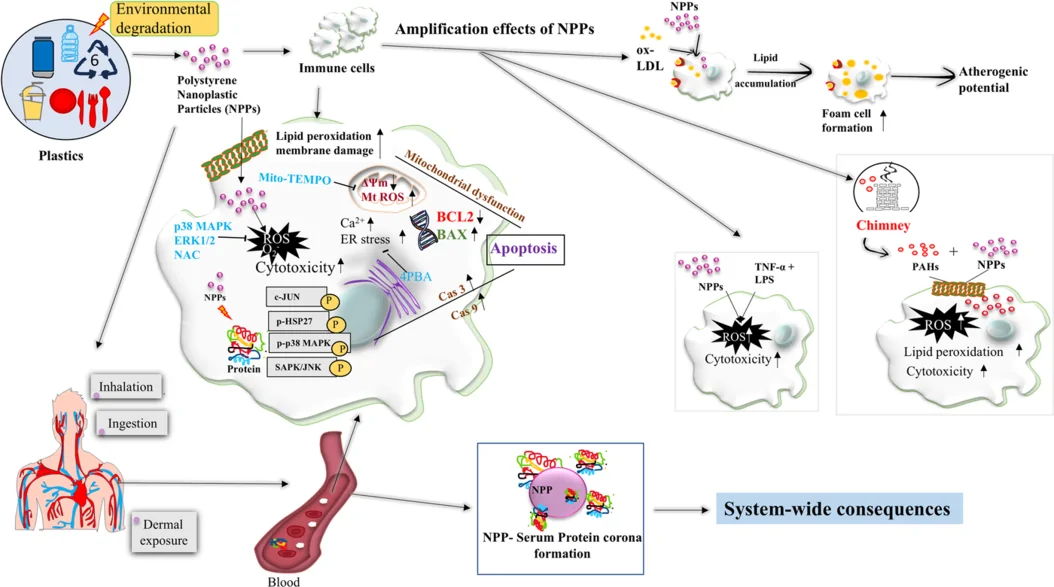

## 1 Introduction

Among the various emerging contaminants, plastic pollution has been a major global problem with annual plastic production increased from 1.5 million tons in 1950 [1, 2] to 413.8 million tons in 2023 [3]. In this regard, polystyrene plastics are extensively used in the manufacturing of packing foods, packing foam, toothbrushes, toys and CDs [4]. These plastics products when released into the environment gradually degraded into microplastics (MPs) (< 5 mm) and nanoplastics (NPs) (< 100 nm) [5] and are been found in soil [6], water [7], air [8], and even in human blood [9]. The nanoplastics due to its smaller size, easily enters into the cells which makes them highly toxic [10] and are exposed through ingestion, inhalation or skin contact [11].

Recent studies revealed that NPPs were found in human blood and also indicated their bioavailability and uptake into the human blood stream [9]. Also, a study carried out among workers exposed to styrene showed evidence of DNA damage [12]. Further, exposure of various cell types (KGN, GES‐1, BEAS‐2B, GC‐1) to NPPs shows oxidative damage interlinked via elevated ROS, disturbance in calcium homeostasis and impaired antioxidant enzymes [13]. Another study indicated that NPPs induce apoptosis by promoting mitochondrial ROS generation, ER stress and inflammation in HK‐2 cells by upregulating p38, Bax, cyt C, and pERK expression [14]. Furthermore, NPPs enhance lipopolysaccharide (LPS)‐induced myocardial fibrosis and autophagy by elevating fibrotic proteins and collagen levels via ROS/TGF‐β/Smad pathway [15].

While focusing on the effect on immune system, which acts as a first line of defense against foreign particles, the TEM, SEM, and live‐cell imaging studies clearly demonstrated concentration‐dependent cytoplasmic uptake of polystyrene micro‐ and nanoplastics by the macrophages [16]. In this regard, NPPs stabilized with anionic surfactant dysregulated the lipid metabolism and mediating the progression of atherosclerosis via macrophage foam cell formation in human and murine macrophages [17]. Further, NPPs promotes necroptosis by mitochondrial ROS generation in macrophages [18] and promotes apoptosis via p38 MAPK activation in zebrafish larvae [19]. Another study illustrates that NPPs induce immunotoxicity in fish by disrupting macrophages and neutrophils synthesis and function [20]. In addition, NPPs exposed to splenic lymphocytes disrupts immune function by inducing apoptosis and downregulating T cell activation [21]. Moreover, considering the ubiquitous presence of polycyclic aromatic hydrocarbon (PAH) in the environment, a previous study illustrated the high affinity nature of plastic particles toward PAHs [22]. Several studies demonstrated that co‐exposure of microplastics and PAHs resulted in oxidative stress, altered lipid metabolism and inflammatory responses in marine bivalve species [23].

Although the impacts of NPPs have been reported in various biological systems, their comprehensive effects on human macrophages and on vulnerable populations (with pathogenic stress and environmental stress conditions) remain unclear. In addition, limited information is available regarding the influence of NPPs on serum protein interaction and corona formation. Addressing these knowledge gaps will improve our understanding of the immunotoxic potential of NPPs under real exposure conditions. Therefore, the current study elucidated the impact of NPPs on serum protein corona formation and explored the cellular and molecular impacts of NPPs on THP‐1 macrophages by evaluating cellular stress and associated signaling pathways. In addition, the study examined the toxicity/pathogenicity amplification potential of NPPs on inflammatory (TNF‐α and LPS), atherogenic (ox‐LDL) and environmental (PAH) stress conditions.

## 2 Materials and Methods

### 2.1 Physical Characterization of NPPs

#### 2.1.1 Size Characterization in Dynamic Light Scattering (DLS)

Particle size distribution measurement was performed using Anton paar lightsizer 500 instrument (Anton paar, Australia). NPPs were diluted to 100 µg/mL and measured in distilled water immediately after 30 min. Values for water properties used for measurement were as follows: refractive index = 1.330, dielectric constant = 78.5 and viscosity at 25°C = 0.89 mPa.s. Particle size was measured as a function of the light scattered by individual nanoplastics which was calculated by the Stokes‐Einstein equation (*R*_H_ = *kT*/6*πηD*), where *k* = Boltzmann constant, *T* = temperature (298 K), and *η* = viscosity of water at 25°C [24]. All size distributions were derived from 10 individual scans.

#### 2.1.2 Scanning Eelectron Microscopy (SEM) Imaging

The morphology of NPPs was analyzed using the Scanning Electron Microscopy (TESCAN—MIRA3 XMU Field Emission SEM) method as described previously [25]. Briefly, 1 μL of sample was placed on a sample holder and air dried. The sample was coated with a thin chromium film using sputtering before analysis.

### 2.2 Chemicals and Reagents

The NPPs utilized in this study (diameter: 100 nm, density: 1.05 g/cm^3^) were commercially procured from Sigma‐Aldrich, USA (Catalogue no: 43302) in the form of an aqueous suspension as described earlier [25, 26, 27]. Reagents including RPMI 1640 culture medium, fetal bovine serum (FBS), cell culture growth factors, caspase‐3, ‐9 substrates, protein kinase pathway inhibitors (FR180204, SP600125, 4‐Phenylbutyric acid, fasudil hydrochloride, AG490, (5Z)‐7‐Oxozeaenol, SB202190, lithium chloride, MitoTEMPO, N Acetyl cysteine (NAC) and PDTC), Phorbol‐1_2_‐myristate‐13‐acetate (PMA), TNF‐α, lipopolysaccharide (LPS), molecular biology grade water and 16 PAHs kit were obtained from Sigma‐Aldrich, USA. Fluorophores employed in cell culture experiments were obtained from Invitrogen, Thermo Fisher, USA. All the other reagents were all commercially accessible and of the highest purity. Where necessary, the experiments used ultrapure water from the Arium pro UV ultrapure water system (Sartorius, Germany).

### 2.3 Cell Culture Conditions and NPPs Exposure

The human monocytic leukemia cell line THP‐1 was acquired from the National Centre for Cell Science (NCCS), Pune, India. Cell lines were grown in RPMI 1640 medium enriched with 10% FBS, penicillin (100 units/mL), streptomycin (100 µg/mL), 0.05 mM 2‐mercaptoethanol (BME) (2 µL) along with appropriate growth factors. Cells were properly maintained in a humidified atmosphere (95% air and 5% CO_2_) at 37°C using an incubator (Nectarnova, Symbiogen, Japan) and passaged when it reaches 80%–90% confluence. Macrophage differentiation was achieved by the addition of 25 nM phorbol 12‐myristate 13‐acetate (PMA) for 48 h and then incubated another 24 h without PMA as described previously [28] before NPPs exposure. The cells were treated with 50, 100, 200, and 400 μg/mL NPPs for assessing cytotoxic effects. Based on viability analysis, 200 μg/mL was selected as the highest concentration that did not significantly reduce cell viability. Hence, subsequent stress related assays were performed by 50, 100, and 200 μg/mL NPPs. Fluorescence based experiments were carried out using tissue culture grade clear bottom black 96 well plates (Invitrogen, USA).

### 2.4 Cellular Impacts of NPPs

#### 2.4.1 Cell Viability Assessment—Resazurin Assay

Resazurin assay was performed to analyze the cell viability/cytotoxicity as described earlier [29]. Mitochondrial enzymes in viable cells reduces the resazurin to fluorescent resorufin, indicating viable cells. Following the treatment, 80 µM resazurin (Sigma‐Aldrich, USA) was added and placed in incubator at 37°C for 4 h. Fluorescence intensity was quantified at 540/590 nm using a BioTek Synergy H1MFG reader. Percentage of cell viability was calculated from fluorescence values (a.u.) relative to control.

#### 2.4.2 Caspase‐3, ‐9 Activity Assay and Apoptosis Validation

Caspase‐3 and ‐9 activities were evaluated using Ac‐DEVD‐pNA and Ac‐LEHD‐pNA substrates, respectively. Hydrolysis of the substrates releases pNA, which was analyzed by measuring absorbance at 405 nm [30] using a BioTek Synergy H1MFG reader. Activities were presented as fold change relative to control. Apoptosis was assessed by a flow cytometry method employing an FITC Annexin‐V/Dead cell apoptosis kit (Invitrogen, USA). Annexin V selectively binds to phosphatidyl serine that is shifted from inner to outer plasma membrane surface during early apoptosis, whereas Propidium iodide (PI) detects late apoptotic, necrotic and dead cells [31]. Concisely, the treated cells were dispersed in 100 µL binding buffer, incubated with 5 µL of Annexin V/FITC and 5 µL propidium iodide at room temperature for 15 min. Cells were subsequently diluted with 400 µL binding buffer and analyzed using BD FACSVerse flow cytometer.

### 2.5 Cellular Oxidative Stress Effects of NPPs

To measure cellular superoxide (O_2_^−^) anions, the cells were loaded with 5 µM DHE (Dihydroethidium) for 30 min at 37°C after treatment [32]. DHE reacts with O_2_^−^ produces red fluorescent products, including 2‐hydroxyethidium or ethidium [33]. Fluorescence was measured at 510/590 nm using a BioTek Synergy H1MFG multimode microplate reader and the data were presented in arbitrary unit (a.u.). To measure the intracellular ROS production level, the cells were incubated with 10 μM H_2_DCFDA (Invitrogen, USA) for 45 min at 37°C in CO_2_ incubator. This probe easily traverses the cells, deacetylated into H_2_DCF by esterases within the cell and gets oxidized into green fluorescent DCF by reactive oxidants [34]. Fluorescence was measured at 485/530 nm using a BioTek Synergy H1MFG multimode microplate reader and the data were presented in arbitrary unit (a.u.) [35]. To measure the lipid peroxidation in live cell membranes, cells were incubated with 1 μM BODIPY 581/591 C11 undecanoic acid lipid peroxidation probe for 30 min at 37°C after treatment. Oxidation of the polyunsaturated butadienyl portion of the probe by reactive lipid derived radicals alters the fluorescence of the probe from red to green Fluorescence was measured at both non‐oxidized (580/610 nm) and oxidized (485/510 nm) forms using a BioTek Synergy H1MFG multimode microplate reader and lipid peroxidation was quantified ratiometrically [29].

### 2.6 Mitochondrial Effects of NPPs

To assess the mitochondrial super oxide generation, cells were incubated with 5 μM MitoSOX Red fluorophore at 37°C for 30 min after treatment as described earlier [36]. This probe specifically accumulates in the energized mitochondria due to the presence of lipophilic methyltriphenylphosphonium cation and gets oxidized by mitochondrial ROS, which results in the generation of red fluorescence The relative signal intensity in cell was quantified at 510/580 nm using a BioTek Synergy H1MFG reader. To estimate the mitochondrial membrane potential, cells were incubated with 100 nM of a cationic and lipophilic dye tetramethylrhodamine ethyl ester (TMRE) at 37°C for 30 min after treatment. TMRE specifically accumulates within mitochondria with high membrane potential. The relative signal intensity in cell was measured at 540/580 nm using a BioTek Synergy H1MFG multimode microplate reader and the data were presented in arbitrary unit (a.u) [37].

### 2.7 Autophagy Evaluation in Cells—AO Staining

The cellular autophagy was quantified by incubating cells with acridine orange (AO) dye [29]. AO emits red fluorescence in the acidic vesicle organelles, while cytoplasm appears as green. The high‐resolution fluorescence microscope (12 MP) (Amscope, USA) was utilized to observe the fluorescence intensity along with cell morphology. The fluorescence quantification was performed from 10 random cell images using ImageJ software.

### 2.8 ER Stress and Protein Aggregation Detection by Thioflavin T

To assess the endoplasmic reticulum stress, cells were incubated with 5 µM of Thioflavin T (ThT) in each well of a 24‐well plate for 30 min at 37°C, following the treatment schedule. ThT specifically binds to protein aggregates [38]. Further, 150 µL of Triton (0.1%) was added in PBS to lyse the cells and 100 µL of the cell lysate was transferred to a 96 well plate for fluorescence analysis at 485/535 nm using a BioTek Synergy H1MFG multimode microplate reader. The fluorescence intensity was normalized to total protein levels.

### 2.9 Cell Imaging Experiments

The microplate‐based assays were validated using the fluorescence imaging experiments for the control and highest dose of exposure (200 µg/mL). Cells grown on glass cover slips placed in 24‐well culture plates were subjected to treatment. Further, fluorophores such as DHE, DCFDA, MitoSOX Red, TMRE, and ThioflavinT were added with appropriate concentrations as described in earlier sections. The high‐resolution fluorescence microscope (12 MP) (Amscope, USA) was utilized to visualize the stained cells by appropriate Ex/Em filters and the fluorescent images were analyzed using ImageJ software [39].

### 2.10 Intracellular Calcium Flux—Fura‐2 AM Assay

The intracellular calcium levels (Ca^2+^) were measured by incubating the cells with a calcium‐binding fluorescent dye, Fura 2‐AM (Sigma Aldrich) [39] (5 µM) at 37°C for 30 min, after treatment. The shift of fluorescence was measured using dual excitation and emission method (Excitation: 340 and 380 nm; Emission: 510 nm) by BioTek Synergy H1MFG multimode microplate reader. The unbound or calcium‐free Fura dye was examined at excitation/emission wavelengths of 380/510 nm while the calcium‐bound dye was evaluated at 340/510 nm. The 340/380 ratio was estimated to determine calcium levels between groups and presented as fold change.

#### 2.10.1 Live‐Cell Ca^2+^ Imaging Using Confocal Microscope

Intracellular calcium accumulation was visualized using Fura‐2 AM dye. Cells were incubated with Fura‐2 AM as mentioned in earlier section and subjected for fluorescent imaging employing confocal laser scanning microscope (Olympus FV3000). The images were obtained using Fluoview acquisition and analysis software. Moreover, image analysis was performed through ImageJ [29].

### 2.11 Western Blotting

Proteins were extracted by lysing the cells in RIPA‐buffer containing protease and phosphatase inhibitors (Sigma Aldrich) and centrifuged at 10,000 × *g* for 20 min at 4°C. The supernatants were collected to estimate the protein concentration based on BCA protein assay method. Total protein content was separated by 10% SDS‐PAGE and the gel was transferred in PVDF‐membrane. The membranes were blocked using 3% BSA and probed with primary antibodies‐ Phospho‐HSP27, Phospho‐SAPK/JNK, Phospho‐c‐Jun, and Phospho‐p38MAPK (Cell signaling technology, USA). GAPDH antibody served as the internal loading control. After HRP‐conjugated secondary antibody incubation and washing, Enhanced Chemiluminescence (ECL)‐reagent was added onto the membrane [40] and imaged using a digital SLR system [41]. Band density was quantified using ImageJ‐software.

### 2.12 Impact of NPPs on Gene Expression

After the study period, THP‐1 cells were subjected to total RNA extraction using GenElute Mammalian Total RNA Miniprep Kit (Sigma‐Aldrich). AMV reverse transcriptase and random hexamers were used for synthesizing cDNA from 1 μg of RNA through reverse transcription. The KAPA‐SYBRFAST‐kit (Sigma Aldrich, USA) was used for real‐time qPCR), with ROX reference dye in a QuantStudio System (Thermo‐Fisher). Relative gene expression of TNF‐ α‐FP: 5′‐ATCAGAGGGCCTGTACCTCAT‐3′ and RP: 5′‐AGACTCGGCAAAGTCGAGATA‐3′; BCL2‐FP: 5′ GAACTGGGGGAGGATTGTGG 3′ and RP: 5′ GCCGGTTCAGGTACTCAGTC 3′; BAX‐FP: 5′ CCCCCGAGAGGTCTTTTTCC 3′ and RP: 5′ CCTTGAGCACCAGTTTGCTG 3′; and ß‐actin (internal reference gene)‐FP: 5′ TCGTGCGTGACATTAAGGAGA 3′ and RP: 5′ ATACTCCTGCTTGCTGATCCA 3′. The PCR product specificity was verified by agarose gel electrophoresis and relative expression levels were analyzed using 2^ΔΔCt^ method [42].

### 2.13 Impact of Pathway Inhibitors and Antioxidants on NPPs Induced Cytotoxicity

As mentioned earlier [42], the cells were pre‐incubated for 2 h with various inhibitors at a fixed concentration as follows: 10 μM ERK1/2 inhibitor (FR180204), 10 μM p38 MAPK inhibitor (SB202190), 10 μM JNK inhibitor (SP600125), 5 mM 4‐Phenylbutyric acid, 10 μM fasudil hydrochloride, 20 mM of lithium chloride (LiCl), 10 μM JAK‐STAT inhibitor (AG490), 1 μM (5Z)‐7‐Oxozeaenol, 3 μM Pyrrolidine dithiocarbamate (PDTC), 1 mM *N*‐Acetyl cysteine (NAC) and 20 μM MitoTEMPO. After preincubation, the cells were exposed to NPPs (400 μg/mL) and cultured in an incubator for 24 h. Subsequent to treatment, cells were exposed to resazurin for assessing cytotoxicity.

### 2.14 Effect of NPPs on Inflammatory Milieu

THP‐1 cells were pre‐treated with NPPs (200 µg/mL) for 2 h. Further, TNF‐α (20 ng/mL, 12 h) was used to induce inflammation related stress in differentiated THP‐1 cells and lipopolysaccharide (LPS‐10 µg/mL, 12 h) was used to induce the inflammation related stress in undifferentiated THP‐1 cells. Cell‐viability and ROS were quantified as mentioned in earlier section.

### 2.15 Impact on Atherogenesis in Macrophages

#### 2.15.1 Atherogenesis—Foam Cell Activation

THP‐1 monocytes were differentiated into macrophages by 160 nM PMA with 10% FBS for 48 h. Then the macrophages were exposed to NPPs (200 μg/mL) for 8 h and subsequently exposed to ox‐LDL (20 μg/mL) (a non‐cytotoxic dose) for 24 h. The oxidant was removed by Hanks’ balanced salt solution before analysis as described previously [43].

#### 2.15.2 Foam Cell Lipid Accumulation—Oil Red O Staining

Oil Red O staining was used to measure the lipid accumulation characteristic of foam cells. After washing, THP‐1 derived macrophages were fixed with 4% formaldehyde for 15 min and stained with Oil Red O (0.3%) for 15 min at 37°C. After isopropanol extraction, the lipid density was measured by absorbance measurement (OD) at 540 nm using a microplate reader (Synergy H1MFG BioTek, USA) as mentioned in previous study [44] and lipid accumulation was visualized under a microscope.

#### 2.15.3 Foam Cell Viability

THP‐1 derived macrophage cells were plated in 96‐well plates and then pretreated with 200 μg/mL NPPs for 8 h and exposed to 20 μg/mL ox‐LDL (a non‐cytotoxic dose) for 24 h. Then, the Resazurin based method was followed as mentioned in earlier section for the evaluation of cell viability [29].

### 2.16 Polyaromatic Hydrocarbons (PAH) Mix and NPPs Co‐Exposure

Cells were co‐treated with NPPs (200 µg/mL) and a PAH mixture (human exposure relevant blood concentration) consisting of 16 Priority PAH with individual concentration based on human blood levels: acenaphthylene (1.45 ng/mL), phenanthrene (37.95 ng/mL), acenaphthene (0.9 ng/mL), naphthalene (45.5 ng/mL), anthracene (58.1 ng/mL), pyrene (33.0 ng/mL), fluoranthene (50.5 ng/mL), fluorene (6.25 ng/mL), chrysene (5.05 ng/mL), benzo(k)fluoranthene (2.45 ng/mL), benz(a)anthracene (16.25 ng/mL), benzo(a)pyrene (1.6 ng/mL), benzo(a)fluoranthene (2.3 ng/mL), dibenzo(ah)anthracene (5.15 ng/mL), benzo(ghi)perylene (5.05 ng/mL), and indeno(1,2,3‐cd)pyrene (4.7 ng/mL), at total concentration of 276.2 ng/mL [45]. Cells were then evaluated for cell viability and cellular superoxide after 24 h of incubation period.

### 2.17 Nanoparticle—Human Serum Protein Interaction (Proteomics Study)

To form the protein corona, 100 µL of NPPs suspension was sonicated and mixed with 100 µL of diluted human serum (1:100). The mixture was incubated for 2 h at 37°C with continual shaking at 200 rpm. After incubation, the samples were centrifuged at 20,000 × *g* for 20 min at 4°C to precipitate the protein corona and the pellets were rinsed 3 times with phosphate buffered saline (PBS) to remove the unbound proteins as detailed in an earlier protocol [39]. The proteins absorbed on NPPs were removed using a gel loading buffer and subject to enrichment through a short gel SDS‐PAGE fractionation method. The gels were cleaned of staining dye and subjected to in‐gel digestion in 50 mM ammonium bicarbonate buffer with 13 ng/µL trypsin and processed as described previously [29]. Protein corona was analyzed by high precision proteomic profiling using liquid chromatography‐mass spectrometry (LC‐MS) [46, 47] using a nano‐ACQUITY UPLC chromatographic system (Waters, Manchester, UK) coupled with a Q‐TOF mass spectrometer (SYNAPT G2 High‐Definition MS System, Waters). Proteins in complex samples were identified by proteomics v4.2 software (Non‐Linear Dynamics, Waters) through Progenesis QI. The peptide sequences were compared against the UniProt database (https://www.uniprot.org/) [39].

### 2.18 Statistical Analysis

GNU‐PSPP statistical analysis software (Boston, USA) was used for statistical analysis. The values were expressed as means ± SD for data interpretation. The current study employed one‐way ANOVA with Tukey‐HSD test and independent *t* test. A *p*‐value of <0.05 was stated as statistically significant.

## 3 Results

### 3.1 Physical Characterization of NPPs

**Figure 1:**
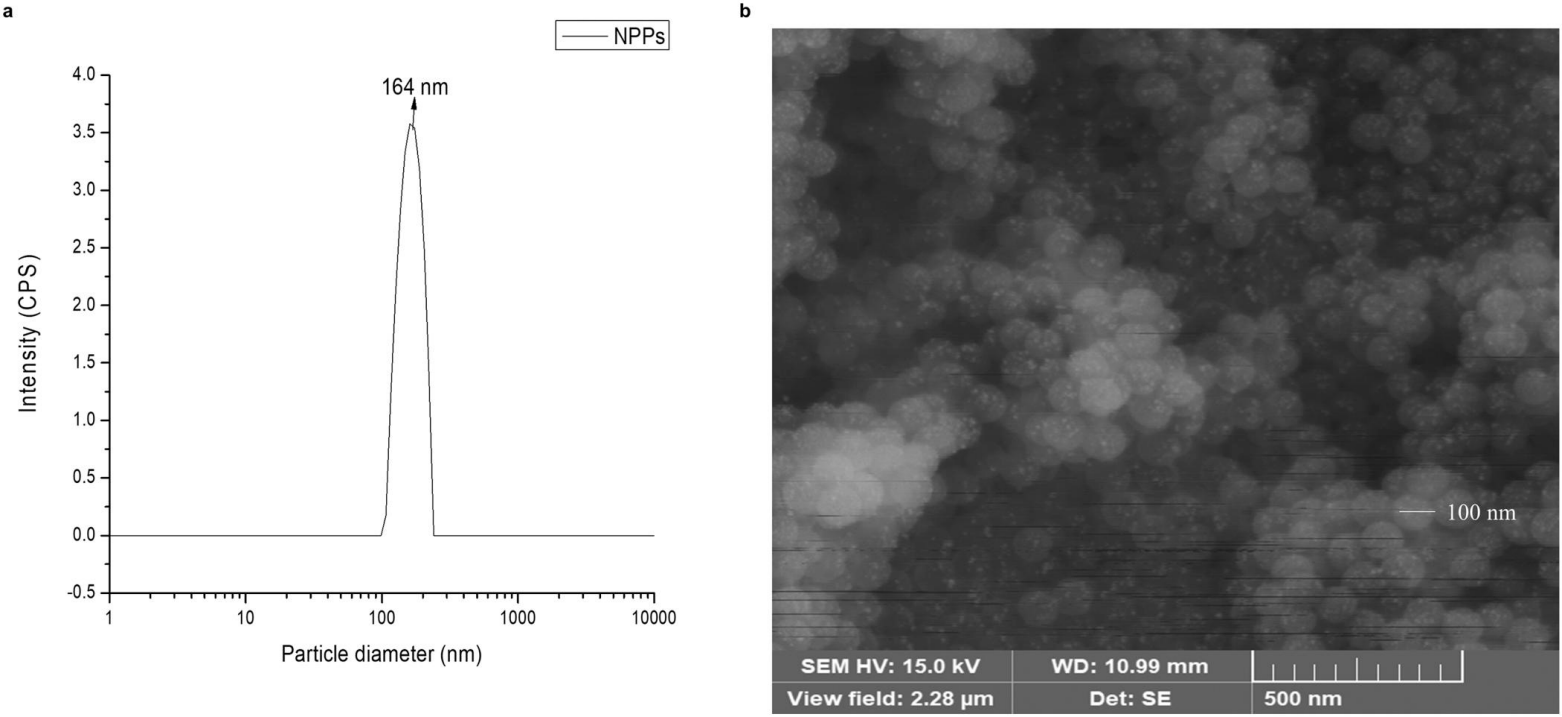
Physical characterization of NPPs. The size distribution analysis of NPPs by DLS (a) and its morphological analysis using SEM imaging (b).

The hydrodynamic size of NPPs was determined using DLS measurements. The average particle size is shown in the Figure 1a and are found to be 164 nm. Further, SEM analysis illustrates that the NPPs are homogenous and with the same size (100 nm). As shown in Figure 1b, the NPPs tend to form a homogenous tridimensional structure and sometimes, due to their physico‐chemical properties (shape, surface charge, size) they clump together.

### 3.2 Effect of Cell Viability and Cytotoxicity

**Figure 2:**
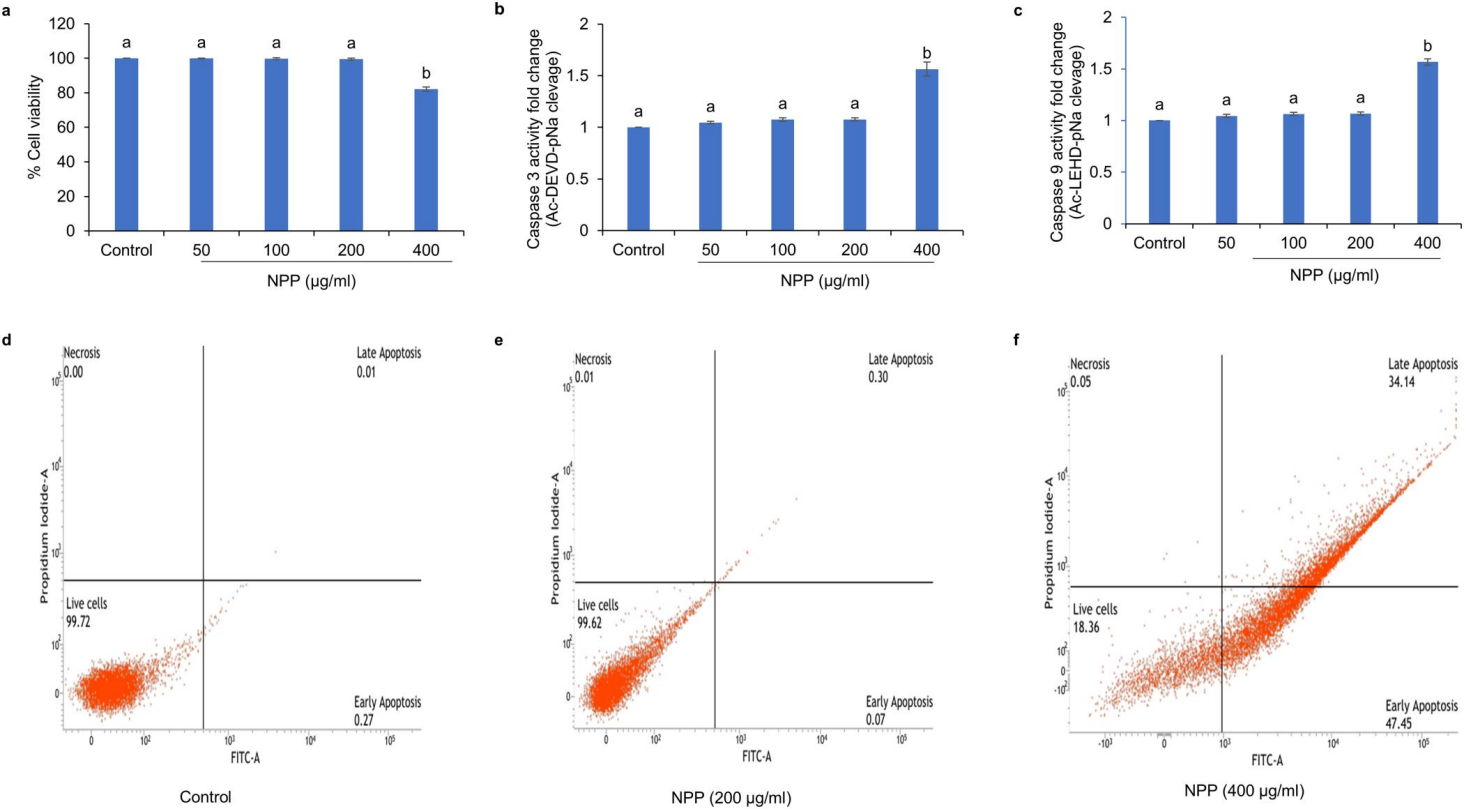
Effect of NPPs on cell viability and cell death. The dose dependent effect of NPPs on cell viability (a), caspase 3 activity (b), caspase 9 activity (c). The flow cytometry analysis of THP‐1 macrophages exposed to polystyrene nanoplastics (NPPs) control (d), cells treated with 200 µg/mL NPPs (e), and cells treated with 400 µg/mL NPPs (f). The represented data are mean ± SD of three identical experiments. Values marked with different letter represent statistically significant differences between each other (*p* < 0.05).

Figure 2a illustrates the dose dependent cytotoxicity of NPPs in THP‐1 derived macrophage cells. The results showed a significant (*p* < 0.05) decrease in cell viability at 400 µg/mL in macrophages when compared to the control and other treated groups (50, 100, and 200 µg/mL).

### 3.3 Apoptotic Cascade—Caspase 3 and 9 Activity Assay

Figure 2b,c shows the dose dependent effects on apoptotic cascade enzymes (caspase 3 and 9) activity in THP‐1 derived macrophage cells, respectively. The results indicate a significant (*p* < 0.05) increase of both caspase 3 and 9 activity at 400 µg/mL NPPs exposure when compared to the control and other treated groups (50, 100, and 200 µg/mL).

### 3.4 Validation of Apoptosis in Cells

The dose dependent cytotoxicity and apoptotic caspase activity results were further confirmed by flowcytometry observation. The flow cytometry results illustrate that NPPs exposure at 400 µg/mL notably elevates both early and late apoptotic (without necrotic) cells when compared with control and 200 µg/mL exposed cells (Figure 2d–f).

### 3.5 Effect of NPPs on Cellular Oxidative Stress

**Figure 3:**
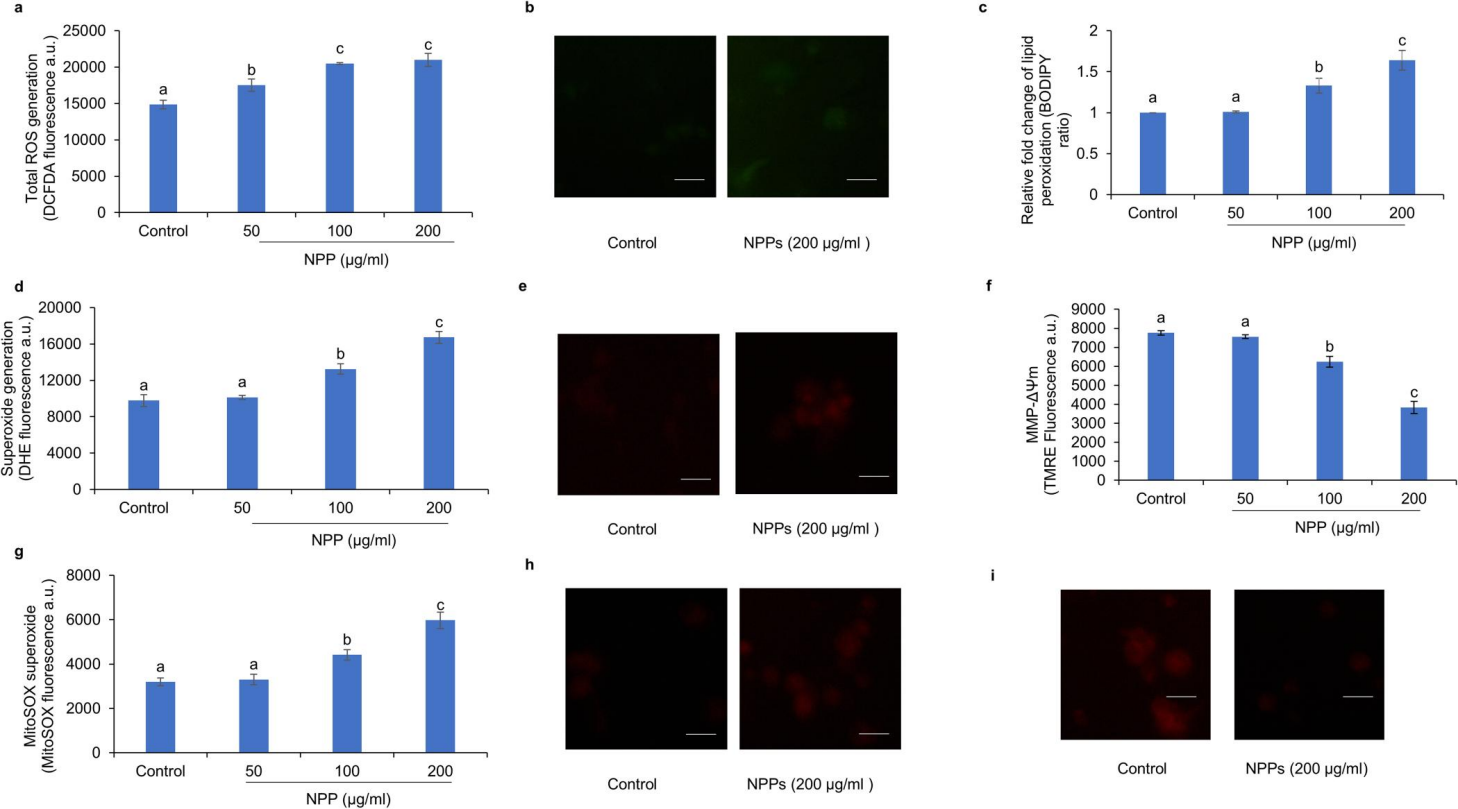
Effect of NPPs on cellular oxidative stress. The dose dependent effect of NPPs on THP‐1 cells: total ROS generation (a), lipid peroxidation (c), superoxide generation (d), mitochondrial membrane potential (f), mitochondrial superoxide generation (g) and the representative fluorescence images corresponding to ROS, superoxide, mitochondrial membrane potential and mitochondrial superoxide are shown in (b, e, i, h) respectively. The scale bar is 100 µm. The represented data are mean ± SD of three identical experiments. Values marked with different letter represent statistically significant differences between each other (*p* < 0.05).

The dose‐dependent effect of NPPs on total ROS, cellular superoxide generation and lipid peroxidation (oxidation ratio of BODIPY C11) in THP‐1 derived macrophage cells were illustrated in Figure 3a,d,c, respectively. The results show that NPPs exposure significantly (*p* < 0.05) increased the total ROS generation at the dosage range of 50, 100, and 200 µg/mL relative to the control. Further, the fluorescent images exhibited increased total ROS and cellular superoxide generation at 200 µg/mL relative to the control (Figure 3b,e), respectively. Notably, cellular superoxide generation and lipid peroxidation were significantly (*p* < 0.05) elevated at 100 and 200 µg/mL (with significant difference between them) as compared to the control and 50 µg/mL.

### 3.6 Impact of NPPs on THP‐1 Cell Mitochondria

The dose dependent (50, 100, and 200 µg/mL) effect of NPPs on cellular mitochondrial superoxide generation and mitochondrial membrane potential (Δψm) in THP‐1 derived macrophage cells were illustrated in Figure 3g,f, respectively. The result indicated a significant (*p* < 0.05) increase of mitochondrial superoxide production and decreased membrane potential at 100 and 200 µg/mL (with significant difference between them) exposed group in relation to control and 50 µg/mL exposed groups. These findings were validated at the cellular level by fluorescence microscopy in cells exposed to 200 µg/mL compared with the control (Figure 3h,i), respectively.

### 3.7 Effect of NPPs on ER Stress and Autophagy

**Figure 4:**
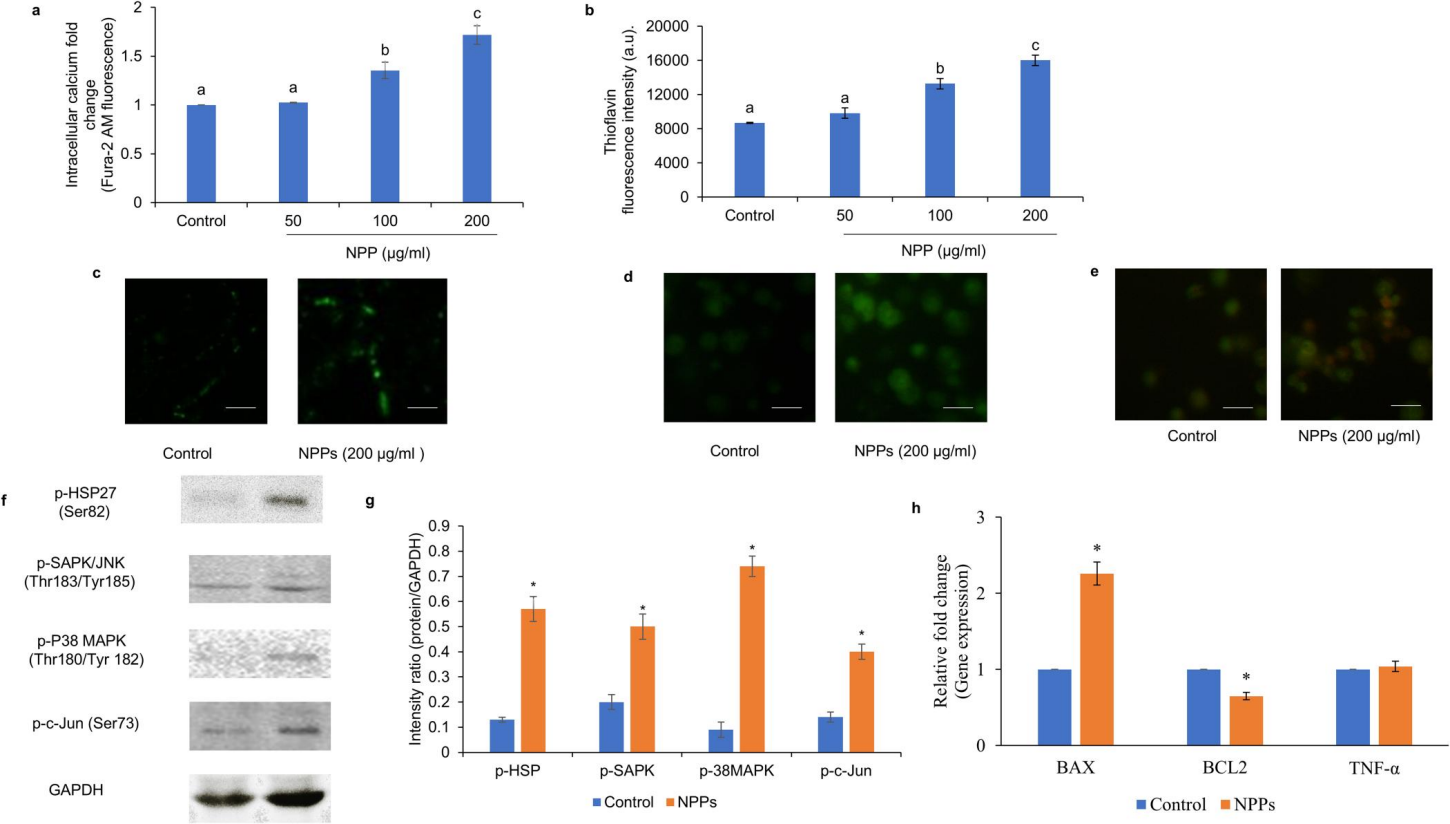
Effect of NPPs on cellular stress, protein and gene expression. The dose dependent effect of NPPs on THP‐1 cells: Intracellular calcium (a), validated by Fura‐2 AM green fluorescence imaging (c) and ER stress (b) with representative fluorescence image (d). Representative fluorescence images showing NPP induced autophagy in THP‐1 cells (e). The scale bar is 100 µm. The represented data are mean ± SD of three identical experiments. Values labeled with different letter indicate statistically significant differences with each other (*p* < 0.05). Representative western blots (f) and relative expression ratios of stress signaling proteins expression (g) in THP‐1 macrophages. The relative fold change of gene expression in THP‐1 cells (h). Values marked with a distinct symbol (*) represent statistically significant differences between each other (*p* < 0.05), whereas, “N.S.” represent statistical non‐significant with each other (*p* > 0.05).

The increasing intensity of ThT fluorescence indicated the unfolded proteins accumulation due to increased ER stress. In the present investigation, a significant (*p* < 0.05) increase of ThT fluorescence was identified at 100 and 200 µg/mL (with significant difference between them) NPPs exposed THP‐1 derived macrophage cells when compared with control and 50 µg/mL (Figure 4b). This finding is validated at the cellular level by fluorescence microscopy in cells exposed to 200 µg/mL compared with the control (Figure 4d).

For autophagy evaluation, acridine orange staining exhibited green fluorescence in the cytoplasm and nucleolus and bright red or orange‐red fluorescence in the acidic compartments. In the present study, the formation of acidic vesicular organelles (AVOs) was evident in THP‐1 derived macrophage cells exposed to NPPs (200 µg/mL) (Figure 4e).

### 3.8 Intracellular Calcium Flux and Microscopic Validation

The intracellular free calcium level indicated by the ratio of calcium unbound and calcium bound signals of Fura‐2 AM dye was depicted in the Figure 4a. The results indicated that NPPs exposure at 100 and 200 μg/mL significantly (*p* < 0.05) elevated the intracellular calcium level (with significant difference between them) compared to control and 50 µg/mL exposed THP‐1 derived macrophage cells. The above result was validated through confocal microscopic images (Figure 4c) and revealed a noticeable elevation of fluorescence signal intensity of Fura‐2 AM dye (Ca^2+^ bound form) in the highest dose (200 μg/mL) of NPP exposed group.

### 3.9 Western Blotting

Figure 4f,g illustrates the representative western blots of phosphorylated proteins and relative expression ratios (bar chart), respectively. The findings demonstrated that phosphorylation levels (activation) of HSP27, SAPK/JNK, p‐38MAPK, and c‐Jun were significantly (*p* < 0.05) increased in NPPs (200 µg/mL) exposed THP‐1 derived macrophage cells in comparison with control.

### 3.10 Gene Expression

Figure 4h illustrate the changes of gene (mRNA) expression in THP‐1 cell during NPPs exposure. The results indicated that NPPs exposure at 400 µg/mL significantly (*p* < 0.05) increased the expression of BAX and decreased the expression of BCL2 against their respective control without any changes in TNF ‐α level (*p* > 0.05).

### 3.11 Effect of Pathway Inhibitors on NPPs Induced Cytotoxicity

**Figure 5:**
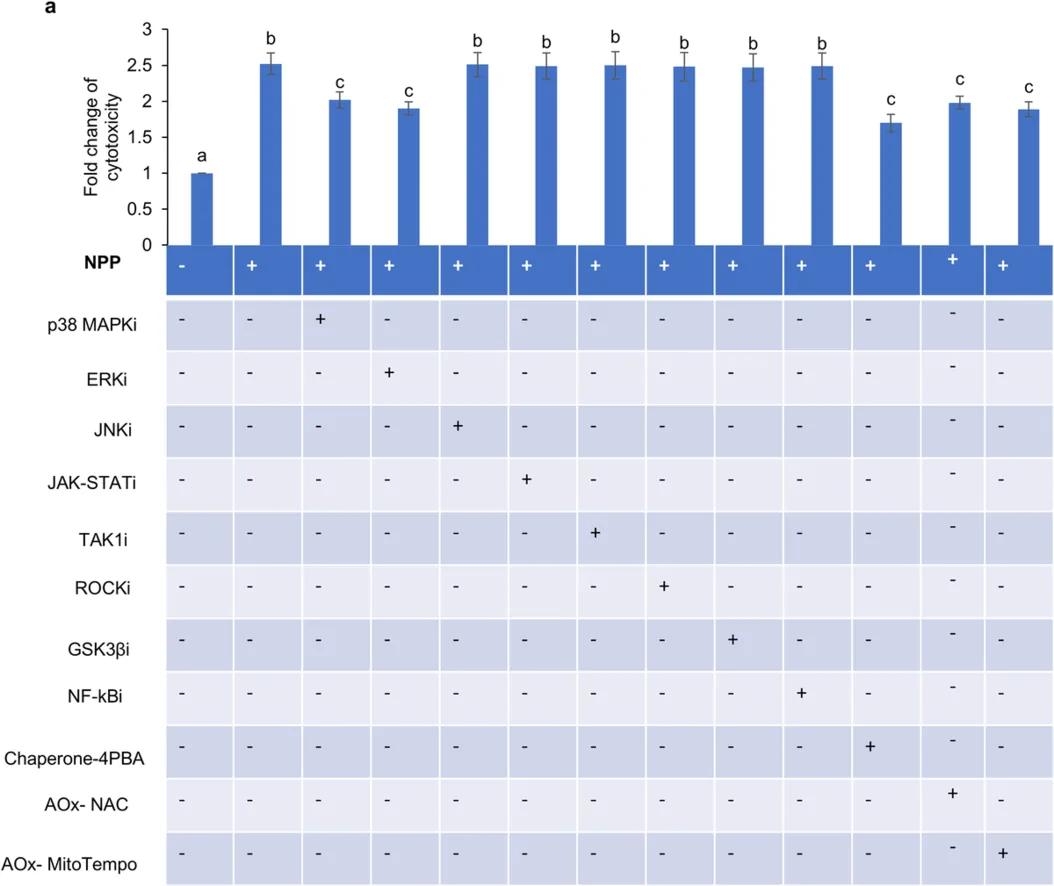
Impact of NPPs on signaling pathways. The impact of pathway inhibitors on cell viability (a). The represented data are mean ± SD of three identical experiments. Values marked with different letter represent statistically significant differences between each other (*p* < 0.05).

The current results on the influence of various pathway inhibitors against of NPPs (400 μg/mL) induced cytotoxicity is illustrated in Figure 5a as relative fold change. The results clearly indicate that NPPs exposure (400 μg/mL) significantly (*p* < 0.05) increased the cytotoxicity. Whereas, the pre‐treatment of THP‐1 derived macrophage cells with kinase inhibitors (p38 MAPK and ERK1/2), chemical chaperone (4‐Phenyl Butyric acid (4‐PBA)) and antioxidants (Mito‐Tempo and NAC) significantly (*p* < 0.05) prevent the cytotoxic response provoked by NPPs exposure.

### 3.12 Stress Amplification Effects of NPPs Under Inflammatory Milieu

**Figure 6:**
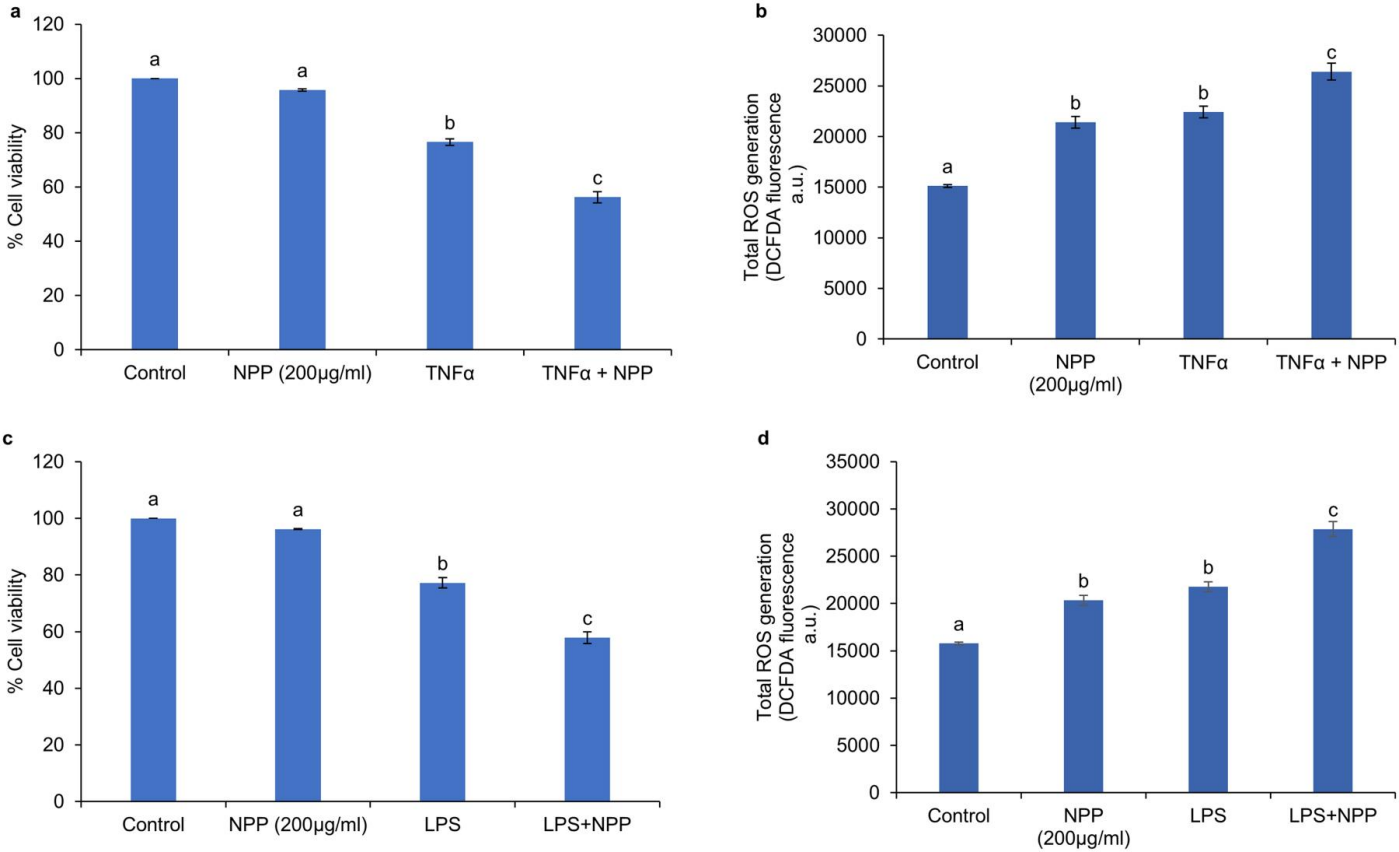
Impact of NPPs on pathological risk factor conditions. The effect of NPPs (200 µg/mL) on cell viability (a) and total ROS generation (b) under TNF‐α simulation and cell viability (c) and total ROS generation (d) under LPS simulation.

The effect of NPPs (200 μg/mL) on cytotoxicity and oxidative stress in THP‐1 macrophage cells under simulated pathogenic/inflammatory milieu by exposing cells with TNF‐α (20 ng/mL) is shown in Figure 6a,b, respectively. In addition, the impact of NPPs (200 μg/mL) on cytotoxicity and oxidative stress in THP‐1 monocyte cells under simulated inflammatory milieu by exposing cells with LPS (10 μg/mL) is illustrated in Figure 6c,d, respectively. Results indicated that NPPs exposure aggravates TNF‐α‐induced cytotoxicity and total ROS generation in a significant (*p* < 0.05) manner in THP‐1 derived macrophage cells in relation to the control, NPP and TNF‐α alone treated groups. Similarly, NPPs exposure aggravates LPS‐induced cytotoxicity and total ROS generation in a significant (*p* < 0.05) manner in THP‐1 monocyte cells relative to the control, NPP and LPS alone treated groups.

### 3.13 Impact of NPPs on Foam Cell Formation (Atherogenesis)

**Figure 7:**
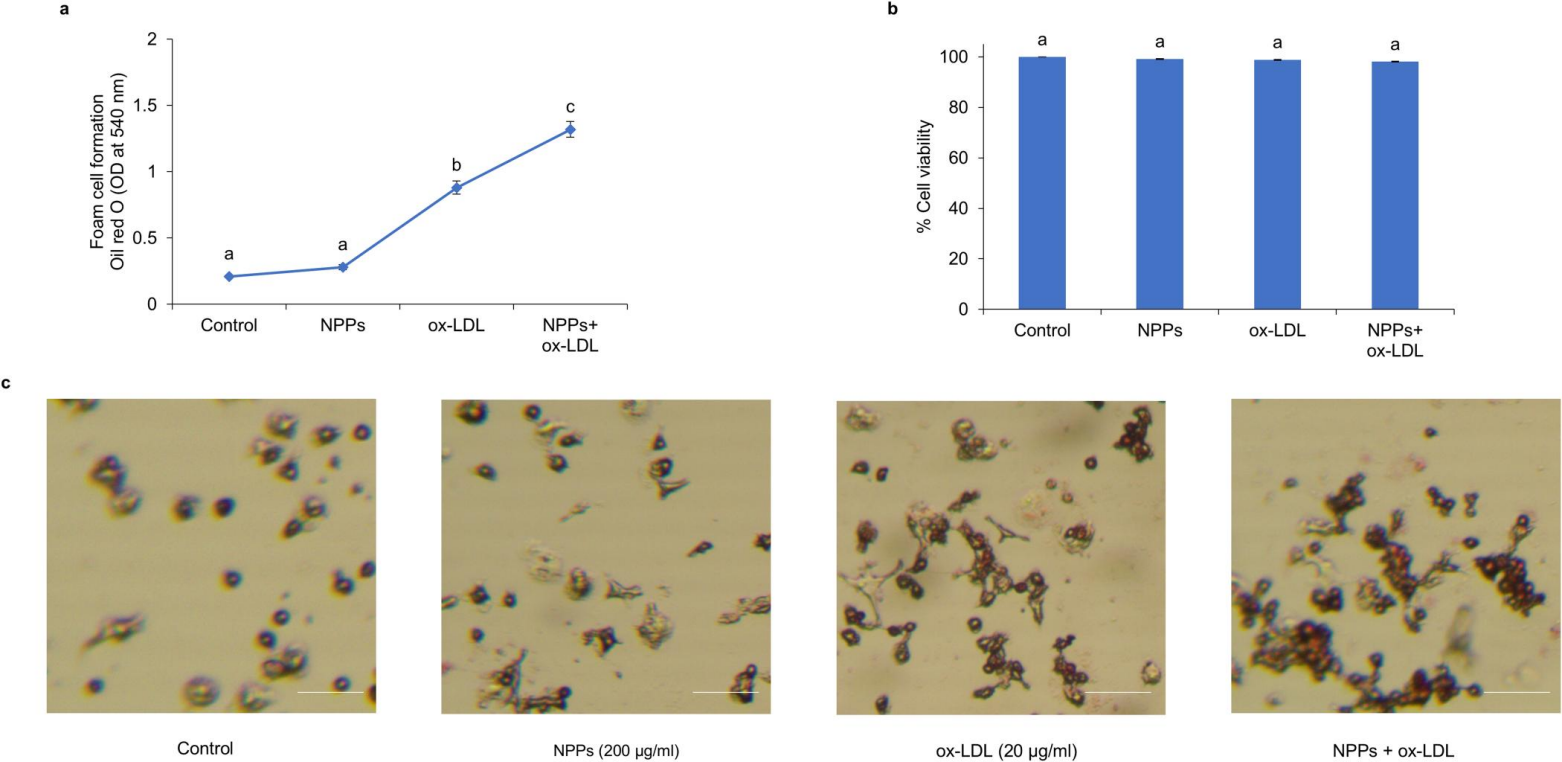
Effect of NPPs on ox‐LDL induced foam cell formation. The impact of NPPs on ox‐LDL induced foam cell formation (a), cell viability (b) and representative oil red O staining images of foam cells (c). The scale bar is 100 µm. The represented data are mean ± SD of three identical experiments. Values marked with different letter represent statistically significant differences between each other (*p* < 0.05).

The impact of NPPs on ox‐LDL induced foam cell formation, cell viability and representative oil Red O‐stained images of foam cells are shown in Figure 7a–c, respectively. The results of OD at 540 nm and oil red O images indicate that NPPs exposure (200 µg/mL) aggravates ox‐LDL (20 µg/mL) promoted foam cell formation (atherogenesis) in a significant (*p* < 0.05) manner in relation to control, NPP alone and ox‐LDL alone treated groups. On the other hand, NPPs exposure does not influence the viability of THP‐1 derived macrophage cells.

### 3.14 Co‐Exposure of NPPs With PAH

**Figure 8:**
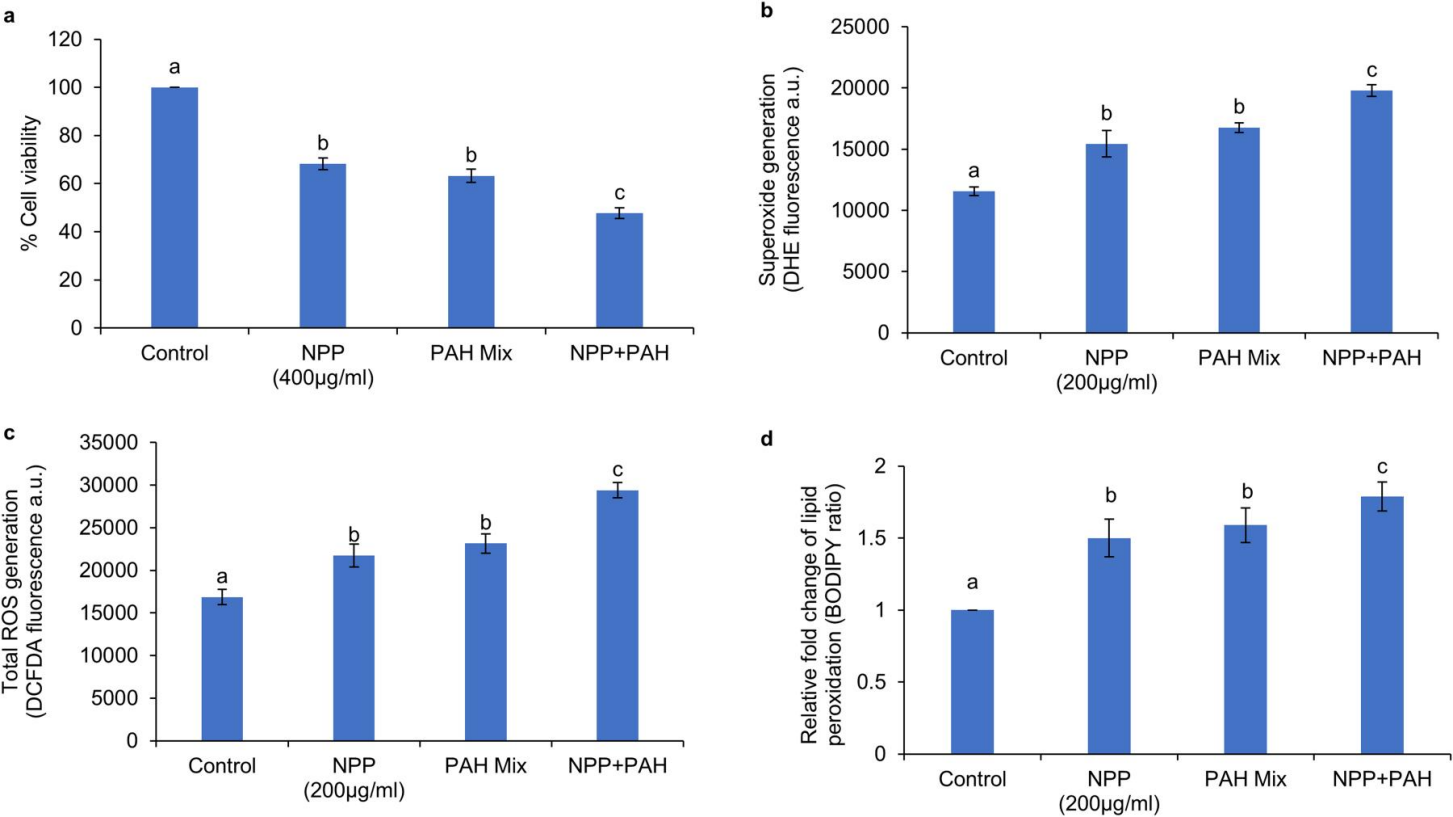
Effect of NPPs co‐exposed with PAH. The impact of NPPs co‐exposed with PAH on cell viability (a), superoxide generation (b), total ROS generation (c) and lipid peroxidation (d). The represented data are mean ± SD of three identical experiments. Values marked with different letter represent statistically significant differences between each other (*p* < 0.05).

The stress amplification effect of NPPs on 16 PAH mixture (human relevant dose) induced oxidative stress in THP‐1 cells is illustrated in Figure 8a–d. Results shown that co‐exposure of THP‐1 cells with NPPs and PAH significantly (*p* < 0.05) aggravates/amplifies cytotoxicity, superoxide generation, total ROS production and lipid peroxidation when compared to the control, NPPs and PAH alone treated groups.

### 3.15 NPPs—Human Serum Protein Corona Composition

The list of proteins present in NPPs‐protein corona (17 unique proteins) is shown in Table 1, notably including immunoglobulin, apolipoprotein, keratin, shroom3, ZNHIT2.

**Table 1:** List of proteins present in NPP‐protein corona.

S. No	Accession	Name
1	P04433	Immunoglobulin kappa
2	P01624	Probable non‐functional immunoglobulin kappa variable 3–7
3	A0A075B6P5	Immunoglobulin kappa variable
4	P0DOX8	Immunoglobulin lambda‐like polypeptide
5	P0DOY2	Immunoglobulin lambda constant
6	P01619	Immunoglobulin kappa variable 3–20
7	P01834	Immunoglobulin kappa constant
8	P02647	Apolipoprotein A‐I
9	P04259	Keratin_ type II
10	P04264	Keratin_ type II cytoskeletal 1
11	P05783	Keratin_ type I cytoskeletal 18
12	P08729	Keratin_ type II cytoskeletal 7
13	P13645	Keratin_ type I cytoskeletal 10
14	P35527	Keratin_ type I cytoskeletal 9
15	P35908	Keratin_ type II cytoskeletal 2 epidermal
16	Q8TF72	Protein Shroom3
17	Q9UHR6	Zinc finger HIT domain‐containing protein 2

The protein corona composition found through LC‐MS/MS.

## 4 Discussion

Macrophages are important cellular component in regulating immune function through phagocytosis [48]. In addition, it was documented that nanoparticles are largely found in macrophages, highlighting the immune system's role in attempting to clear the nanoparticles from the system [17]. The present study assessed the impact of NPPs on THP‐1 macrophages through extensive experimental protocols. Initially, the dose‐dependent effect of NPPs on cell viability and apoptosis were analyzed. In this regard, studies stated that nanoplastic exposure on murine preosteoblasts, osteoblasts and preosteoclasts affect the cell viability through ROS formation and apoptosis (through caspase3/7) [49]. Apoptosis can occur through intrinsic and extrinsic pathways, where extrinsic pathway is induced by external stimuli, activating caspase 3 and promoting cell death [50] whereas intrinsic pathway involves loss of mitochondrial membrane permeability, cytochrome c release, caspase 9 activation and ultimately leads to caspase 3 activation and cell death [51]. Moreover, a recent study illustrated that apoptosis initiated in zebrafish through oxidative stress mediated caspase 3 activation [52]. The results illustrate that NPPs exposure induced a dose‐dependent increase in cytotoxicity, accompanied by significant elevation of caspase 3 and caspase 9 in THP‐1 cells and flow cytometry results further verified the induction of both prominent early and late apoptosis in NPP exposed cells. Although 200 µg/mL NPPs did not cause a significant decrease in cell viability, this concentration has activated multiple stress pathways. Thus, the NPPs exposure toxicity needs consideration.

In addition, the evaluation of oxidative stress, a state of imbalance of pro‐oxidant and anti‐oxidant species [53], shows that NPPs increased the total ROS generation, superoxide anion and lipid peroxidation in THP‐1 macrophages. In this context, previous study reported that excessive oxidative stress triggers lipid membrane peroxidation, damage to DNA base and DNA and protein cross‐links, which leads to apoptosis, necrosis, inflammation and mitochondrial dysfunction [54]. Further, oxidative stress is known to cause lipidome wide changes through lipid peroxidation and consequently leads to adverse cellular effects [55]. Another study also illustrates that NPPs can induce lipid peroxidation by increased mitochondrial ROS generation in human and murine macrophages [17]. A recent study revealed that polystyrene microplastics disrupts mitochondrial structure through oxidative stress in mouse GC‐2 cells [56]. Also, imbalance in redox status of the cell leads to pathophysiology of various disease outcomes [57]. Following, another study has documented the intensifying effect of NPPs on mitochondrial ROS generation and stimulated mitochondrial dysfunction by promoting necroptosis in mice and human macrophages [18]. In this regard, the observed results indicated the NPP induced mitochondrial ROS production and depolarization of the membrane potential (mitochondrial dysfunction) in THP‐1 macrophages. Therefore, mitochondrial dysfunction and oxidative stress play a major role which may provide novel mechanistic perceptions on NPPs induced macrophage cell toxicity.

The impact of NPPs on endoplasmic reticulum (ER) stress and intracellular Ca^2+^ level in THP‐1 macrophages were also studied and found notable variations in these processes. As mentioned in the previous section, NPPs exposure elevates ROS generation. In this regard, there are close connections between oxidative stress and Ca^2+^ homeostasis where high oxidative conditions trigger dysregulation of Ca^2+^ homeostasis [58]. Consequently, disruption of Ca^2+^ homeostasis promotes ER stress [59]. In addition, a recent study reported that NPPs exposure causes lung damage through ROS‐dependent ER stress in BEAS‐2B lung epithelial cells [60]. Previous study demonstrated that polystyrene microplastics exposure triggered oxidative stress and ER stress in juvenile rats, which leads to inflammation and renal apoptosis [61]. ER stress disturbs cell homeostasis, induces apoptosis and enhances foam cell formation and pro‐inflammatory cytokine production in macrophages [62]. The M1 polarization enhancement and concurrent suppression of M2 polarization in response to targeted electric signal were mainly because of Ca^2+^ influx via voltage‐gated channels [63]. Acridine orange is a cell‐permeable green fluorophore that can be protonated and trapped in acidic vesicular organelles (AVO) which is a quick, accessible and reliable method to assess the volume of AVOs, which increases upon autophagy induction [64]. The observed results indicate increased ER stress and autophagy and elevated intracellular Ca^2+^ level (calcium accumulation) may mediate via oxidative stress, thereby increasing the attention towards its role during risk assessment of NPPs.

Notably, western blotting unveiled that NPPs elevated the phosphorylation of HSP27, SAPK/JNK, p38 MAPK and c‐Jun expression in THP‐1 macrophages. Heat shock protein 27 (HSP‐27) is a protein that has chaperone, antioxidant and anti‐apoptotic properties and is induced in response to stress [65]. Recent evidences indicate that NPPs elevated HSP‐27 expression in human neural stem cells [66]. In addition, SAPK/JNK, c‐JUN, p38 belongs to mitogen‐activated protein kinases (MAPKs) in mammals plays necessary role in regulating cell proliferation, apoptosis, oxidative stress, inflammation and autophagy [67]. Consistent with the observed results, a study reports that immune dysfunction was induced by NPPs via MAPK pathway [68]. Another study has documented the involvement of p38 and JNK pathways during Al_2_O_3_‐NPs nanoparticle induced oxidative distress [69]. c‐Jun overexpression was observed in many kinds of autoimmune diseases [70]. Therefore, the current study revealed the plausible cell stress conditions and adaptive responses by THP‐1 cells via increased phosphorylation of various cell stress signaling proteins, which in turn activate its signaling pathways during NPPs exposure.

While focusing on apoptosis and inflammation related expression of gene, the BCL2 family proteins is vital for organismal development, tissue homeostasis and precisely regulate apoptosis. Further, the BCL2 associated X protein (BAX) is a key factor in mitochondrial mediated cell death [71]. Apoptosis (programmed cell death) is determined by the BAX/BCL2 protein ratio, where a higher ratio promotes apoptosis and a lower ratio inhibits it [72]. In this connection, previous research has shown that NPPs and triclosan coexposure profoundly enhanced apoptosis by elevated cleaved caspase‐3 protein expression and reduced the BCL2/BAX ratio [73]. In addition, polystyrene nanoplastics and okadaic acid coexposure significantly increased the number of BAX/BCL2 homodimers in cells and promotes the apoptosis via the release of cytochrome c and other substances and activate caspase‐9 and 3 through the mitochondrial pathway [74]. Further, it has already been revealed that interleukin‐6 (IL‐6) and tumor necrosis factor alpha (TNF‐ α) expression is significant in cardiovascular inflammatory response [75]. Previous study revealed that microplastics exposure triggers the secretion of both inflammatory cytokines (IL‐6, TNF) and anti‐inflammatory cytokines (IL‐10) in primary human monocytes potentially as a feedback mechanism [76]. Other studies have also indicated that MPs and NPPs in vitro exposure changed the expression of other prominent inflammatory markers, including TNF‐ α, IL‐1β, and IL‐8 [77]. In this connection, the current study indicates that at 400 µg/mL dosage exposure elevates BAX expression and decreases BCL2 expression illustrates the involvement of mitochondrial pathways during apoptosis. Alternatively, the expression of TNF‐α indicates that, NPPs may not have direct effect on inflammatory response in THP‐1 macrophage cells which require further validations with other inflammatory mediators such as IL‐1β, IL‐6, IL‐8, MCP‐1, etc.

Furthermore, the pathway inhibitor study indicates that p38 and ERK inhibitors, chemical chaperone (4PBA) and antioxidants (NAC and mitoTEMPO) show protective effect against NPPs induced cytotoxicity. In this regard, an earlier study documented that NPPs induce p38 MAPK activation and apoptosis by eliciting oxidative stress in macrophage cells and zebrafish [19]. Also, ERK1/2 belonging to MAPK family is known to associated with cytokine production [78]. Consistent with previous findings, ERK inhibition significantly attenuates NPP‐induced cellular toxicity [79]. As a chemical chaperone, 4‐phenyl butyric acid (4‐PBA) inhibits ER stress by promoting protein refolding [80], reducing Ca^2+^ levels and inhibiting the expression of the IRE1/XBP1 ERS pathway and apoptotic factors during NPPs and LPS combined exposure in HEK293 cells [81]. Further, various studies illustrate that Mito‐TEMPO, a mitochondria‐targeted antioxidant, stabilize mitochondrial membrane potential, thereby, preventing autophagy and attenuated glutamate‐induced cytotoxicity in neuroblastoma cells [82] and reduced M1 polarization mediated foam cell formation [83]. Similarly, a recent study indicate that mito‐TEMPO reduced the NPPs induced mtROS generation in RAW 264.7 cells [18]. *N*‐Acetylcysteine (NAC), a membrane‐permeable cysteine precursor, a potent antioxidant, a ROS scavenger [84] and anti‐inflammatory agent [85] mainly inhibiting the expression of inflammatory cytokines in macrophages [86]. Concomitantly, NAC alleviated AgNPs mediated cell apoptosis by reducing ROS generation, mitochondrial and lysosomal injury, improving autophagy flux, preventing caspase‐3 activation in HUVEC cells [87]. The exploration revealed the involvement of various kinase signaling pathways and oxidative stress mechanism in the cytotoxic effect of NPPs which was alleviated by inhibiting specific kinases and via administration of antioxidants.

The effect of nanoplastic exposure on individuals with preexisting inflammatory milieu (simulated by in vitro exposure of TNF‐α and LPS) needs further investigation. In this regard, the current results illustrate that NPPs co‐exposure aggravates cytotoxicity and increased the total ROS production in TNF‐α and LPS exposed macrophage and monocyte cells respectively. TNF‐α is a cytokine expressed by macrophages which play a vital role in regulation of inflammation and immune responses against infection and external stimuli [88]. Notably, in various cardiovascular pathogenic condition the TNF‐α level was found to be increased in expression [75]. Further, NPPs exposure aggravated LPS induced necroptosis, immune disorders and inflammatory damage to the mice [89]. Thus, the aggravation potential of NPPs on inflammatory conditions needs further attention during risk assessment in vulnerable population with systemic inflammatory stress.

While emphasizing NPPs' atherogenic aggravation potential during ox‐LDL‐induced foam cell formation, a recent study highlights that microplastics contribute to atherosclerotic risk elevation and increased vascular complexity in acute coronary syndrome patients [90]. Foam cells are formed during macrophages infiltrate into the intima of the arterial wall and uptake excessive lipids and this process is a crucial event during atherogenesis [91]. In this regard, cell absorption experiments indicated that macrophages quickly absorb NPPs of 50 and 500 nm size in a time‐dependent manner [92]. Further, a previous study established that silica nanoparticles exacerbate ox‐LDL‐induced lipid accumulation in macrophages and apoptosis through ER stress [93]. Considering this, the current study indicates the elevated lipid accumulation during NPPs’ co‐exposure with ox‐LDL without cytotoxicity. Hence, considering the enhancement effect of NPPs on atherogenesis is recommended during the risk assessment of NPPs exposure in CVD‐prone populations, especially with dyslipidemia.

Exposure to PAHs occurs through many routes, including ingestion of contaminated food and water and inhalation of contaminated air is linked with range of adverse health effects [94]. In this regard, an earlier study explored that co‐exposure to MPs and PAHs, is more toxic compared to single exposure in marine bivalve species [23]. In this connection, another study illustrates that combined exposure to PSMPs and benzo [a] pyrene (a PAH compound) enhances liver damage by inducing altered oxidative stress, inflammatory responses and lipid metabolism in liver [95]. Subsequently, the observed results revealed that NPP and PAH co‐exposure aggravates oxidative stress and lipid peroxidation mediated cytotoxicity. Hence, the vulnerable populations lived at close proximity to environmental PAH exposure (highly polluted area) may multiply the health risk which needs more consideration during NPPs exposure.

The current proteomic study reveals that NPPs interact with human serum proteins. This interaction forms a protein corona comprising 17 unique proteins, including immunoglobulins, apolipoprotein A1, keratin proteins, Shroom3 and ZNHIT2. Immunoglobulins are the essential protein components of the immune system that identify and bind antigens, which leads to the regulation of innate and adaptive immune responses. It is composed of two identical heavy chains and two identical light kappa or lambda chains. Immunoglobulin light chain is crucial for structural stability, antigen interaction and the regulation of autoantibody [96]. Besides intact immunoglobulin, B cells also release excess free light chains into the bloodstream, contributing to immune regulation [97]. Further, a previous study indicated that the changes in the expression of immunoglobulin lambda constant 1 (IGLC1) are linked with tumor progression and immune evasion [98]. Apolipoprotein AI (ApoAI), a major structural and functional protein of high‐density lipoprotein [99]. It plays a pivotal role in cholesterol reversal transport and exerts significant anti‐inflammatory response by mediating the immune cell function [100]. Structural proteins like keratins maintains mechanical stress, mechanical elasticity, cytoskeletal stability and integrity, cellular morphology, cell signaling, thereby influencing structure and mitotic activity [101]. In addition, Shroom3 is involved in maintaining renal function, cardiac development, gut tube morphogenesis [102] and regulating actomyosin organization [103]. Similarly, ZNHIT2 is a part of the zinc finger histidine triad (HIT) domain family [104], which participates in the assembly of U5 snRNP and RNA processing complex [105]. Overall, the current findings suggest that the adsorption of immune, metabolic, cytoskeletal and regulatory proteins on the surface of the nanoplastic may modulate immune responses, cellular organization and regulatory pathways, providing important insights into the biological and pathophysiological impacts of NPPs during health risk assessment.

## 5 Conclusion

The increasing plastic pollution and its inevitable corresponding exposure, this study emphasizes the need for understanding the underlying mechanisms of nanoplastics toxicity. The present research demonstrates that NPPs exposure triggers macrophage toxicity through ROS mediated mechanism involving oxidative stress, mitochondrial dysfunction and activation of stress signaling pathways. NPPs exposure significantly elevated intracellular and mitochondrial ROS level, lipid peroxidation, calcium signaling, ER stress, autophagy and apoptotic cell death. Pathway inhibition study confirmed the involvement of p38 MAPK, ERK, ER stress and mitochondrial ROS signaling, while activation of HSP27, SAPK/JNK, p38 MAPK and c‐Jun phosphorylation elucidated the mode of action (MoA) during NPPs induced macrophage toxicity. In addition, NPPs exposure shows aggravation potential on inflammation stress, atherogenesis and exacerbated toxic effects under co‐exposure with human exposure relevant concentration of 16 priority PAH mixture. Serum corona profiling demonstrated the initial nano‐bio interaction that may influence the downstream toxicological responses of NPPs. Collectively, these findings provide a valuable understanding on mechanistic insights on immunotoxicity and potential health risks associated during NPPs exposure.

## Author Contributions

Jeganathan Manivannan conceived and designed the experiments. Balamurali Mahalakshmi performed the experiments. Balamurali Mahalakshmi, Gobichettipalayam Balasubramaniam Maadurshni and Thittumpuram Manikandan Anjana analyzed the data. Jeganathan Manivannan validated the data. Jeganathan Manivannan, Balamurali Mahalakshmi and Gobichettipalayam Balasubramaniam Maadurshni wrote the paper. All authors read and approved the manuscript.

## Funding

The authors have nothing to report.

## Conflicts of Interest

The authors declare no conflicts of interest.

## Data Availability

The data that support the findings of this study are available from the corresponding author upon reasonable request.
